# Factors influencing the time-intensity curve analysis of contrast-enhanced ultrasound in kidney transplanted patients: Toward a standardized contrast-enhanced ultrasound examination

**DOI:** 10.3389/fmed.2022.928567

**Published:** 2022-08-25

**Authors:** Sarah Friedl, Ernst Michael Jung, Tobias Bergler, Hauke C. Tews, Miriam C. Banas, Bernhard Banas, Franz Josef Putz

**Affiliations:** ^1^Department of Nephrology, University of Regensburg, Regensburg, Germany; ^2^Department of Radiology, Interdisciplinary Ultrasound, University of Regensburg, Regensburg, Germany; ^3^Department of Internal Medicine I, Gastroenterology, Hepatology, Endocrinology, Rheumatology and Infectious Diseases, University Hospital, Regensburg, Germany

**Keywords:** TIC-analysis, ROI, region of interest, CEUS, contrast-enhanced ultrasound, kidney transplantation, perfusion analysis

## Abstract

**Background:**

Time-intensity curve analysis (TIC analysis) based on contrast-enhanced ultrasound (CEUS) provides quantifiable information about the microcirculation of different tissues. TIC analysis of kidney transplantations is still a field of research, and standardized study protocols are missing though being mandatory for the interpretation of TIC parameters in the clinical context. The aim of this study was to evaluate the impact of different sizes and forms of regions of interest (ROIs) on the variance of different TIC parameters and the level of interoperator variance between the different ROI methods in kidney transplantations.

**Methods:**

In 25 renal transplanted patients, 33 CEUS of the transplanted kidney were performed, and TIC analysis with ROIs sized 5 mm^2^ (ROI_5_), 10 mm^2^ (ROI_10_), and ROIs circumscribing the outlines of anatomical regions (ROI_*Anat*_) were analyzed based on CEUS examination. The TIC analysis was repeated by a second independent operator for ROI_5_ and ROI_*Anat*_.

**Results:**

Statistical analysis revealed significant differences between TIC parameters of different ROI methods, and overall, the interoperator variance was low. But a greater ROI surface (ROI_10_) led to higher values of the intensity parameters A and AUC compared with ROI_5_ (*p* < 0.05). The difference in the ROI form led to high variation of certain TIC parameters between ROI_5_ and ROI_*Anat*_ in the myelon [intraclass correlation coefficient (A, ICC = 0.578 (0.139–0.793); TIC parameter (TTP); and ICC = 0.679 (0.344–0.842) (*p* < 0.05)]. A mean variation of 1 cm of the depth of ROI_5_ in the cortex did not show significant differences in the TIC parameters, though there was an impact of depth of ROI_*Anat*_ on the values of TIC parameters. The interoperator variance in the cortex was low and equal for ROI_5_ and ROI_*Anat*_, but increased in the myelon, especially for ROI_*Anat*_. Furthermore, the analysis revealed a strong correlation between the parameter AUC and the time interval applied for the TIC analysis in the cortex and myelon (*r* = 0.710, 0.674, *p* < 0.000).

**Conclusion:**

Our findings suggest the application of multiple ROIs of 5 mm^2^ in the cortex and medulla to perform TIC analysis of kidney transplants. For clinical interpretation of AUC, a standardized time interval for TIC analysis should be developed. After the standardization of the TIC analysis, the clinical predictive value could be investigated in further studies.

## Introduction

Kidney transplantation is the treatment of choice for patients with end-stage renal disease besides various dialysis procedures (1). Compared with dialysis, patients after successful kidney transplantation benefit from a better quality of life, a higher functional level, and show longer survival (2, 3). With the eldering of society and advanced medical care, the mismatch between organ demand and availability is increasing. In this context, it is important to maintain the function of the allograft as long as possible. The main reason for long-term allograft loss is a combination of immunological and different non-immunological factors (4). In the context of immune responses, inflammation and degenerative changes occur and lead to changes in microcirculation and limitation of allograft function (5). Chronic allograft nephropathy often starts developing within the first year post-transplantation, (6) and until recently, the invasive biopsy is the gold standard for diagnostics. However, the utility of protocol biopsies is useful to determine the degree of chronic damage but is discussed controversially because of their invasive nature and is not performed in every transplant center (7). Recently, more and more progress was made in non-invasive methods to assess transplant function. In this study, especially, biomarkers in serum (8) and urine (9) have been developed. In the field of apparative diagnostics, there is a focus on modern MRI techniques (10) and CT perfusion imaging (11).

Contrast-enhanced ultrasound (CEUS) allows the description of the microcirculation of organs and is more and more used in the examination of kidneys and kidney transplants. Time-intensity curve analysis (TIC analysis) in kidney transplantation is a novel technique of perfusion analysis, and there are promising data that TIC analysis could provide useful information to determine the prognosis of allograft early and non-invasively (12, 13). TIC analysis allows the objective measurement of the contrast kinetics within a defined region of interests (ROIs) and therefore describes the microcirculation. Based on CEUS examination, perfusion parameters are calculated using integrated or external software that applies a perfusion model in a selected ROI in the kidney. The advantages of CEUS are its availability, low cost, and safe application without nephrotoxic effects, so this technique can be applied to a broad mass of patients, especially as chronic kidney disease is no contraindication in comparison to other perfusion imaging modalities, such as contrast-enhanced CT scans (14). Currently, results of TIC analysis are only comparable to a limited extent, and TIC analysis in kidney transplants is still considered a field of research (7). Numerous factors such as instrument settings during CEUS examination, application of contrast medium, patient-related data (i.e., blood pressure and body mass index), and different analysis software have been shown to influence perfusion parameters (15–17). Although TIC analysis is an emerging field of research, there is neither clarification about the impact of size, form, and localization of the different ROIs nor do we know much about the interoperator variance of TIC analysis.

In this study, we evaluated different methods of TIC analysis in renal transplantations and compared different factors influencing the quality of the measurement parameters (e.g., depth of the kidney and length of the cine loop). By repeating the measurements by another investigator, we checked the interreader variance. The aim of this study was to develop a standardized TIC analysis protocol with low intraoperator and interoperator variance and high feasibility.

## Materials and methods

### Patients and contrast-enhanced ultrasound examination

Between May 2017 and January 2019, 25 patients aged from 22 to 79 years with kidney transplants (mean organ age since KTx 5.18 years) received 33 CEUS at the University Hospital Regensburg by an experienced sonographer. Kidney-transplanted patients (>18 years) with a stable graft function and a CEUS examination suiting the study protocol were included in the study. Patients with pathologies of the transplanted kidneys (e.g., infarction, renal artery stenosis, infection, and ureteral obstruction) and patients with unstable hemodynamics were excluded from the study. In addition, CEUS studies that did not meet the quality requirements for the subsequent TIC analysis (e.g., stable image and length) were also excluded. There were no significant differences in the hemodynamics (e.g., blood pressure and cardiac function) of the patients.

Before CEUS, a complete status of the transplanted kidney was obtained including a B-mode scan and color-coded Doppler sonography. CEUS was performed in the “low-MI technique” (MI, mechanical index) with MI values < 0.09 (12). The setting of depth, gain, and focus was adjusted to the optimal display, with focus at the deepest point of the transplant. After giving written informed consent, patients received a 1.5 ml bolus of ultrasound contrast agent (sulfur hexafluoride microbubbles, SonoVue^§^, Bracco, Italy) followed by a 10 ml saline flush *via* intravenous administration in the cubital vein. After the injection of the contrast agent was completed, a timer was started. All examinations, including TIC analysis, were stored digitally (DICOM format). CEUS examination and data collection were permitted by the Ethical Committee of the University of Regensburg (17-662-101_P1, 17-662-101_P2, and 17-662-101_P3).

### Time-intensity curve analysis

TIC analysis was performed based on 33 CEUS examinations by two operators separately. Both operators were blinded to the clinical parameters and the transplant outcomes. To check the robustness of the investigation and the ease of application, the investigations were carried out by two operators with different levels of experience. Operator 1 was an advanced medical student, and Operator 2 was a nephrologist experienced in the field of CEUS. The analysis was carried out using the integrated software of Logiq E9 (GE Healthcare, United States). A mathematical model for typical “Wash-in” kinetics was used for curve fitting. The starting point of TIC analysis was set at the arrival of the contrast agent in the central artery of the kidney (18), and the end of TIC analysis was set after 60 s on average or TIC analysis was determined earlier by the end of the video clip. We applied three different methods of ROI to perform the TIC analysis.

**FIGURE 1:**
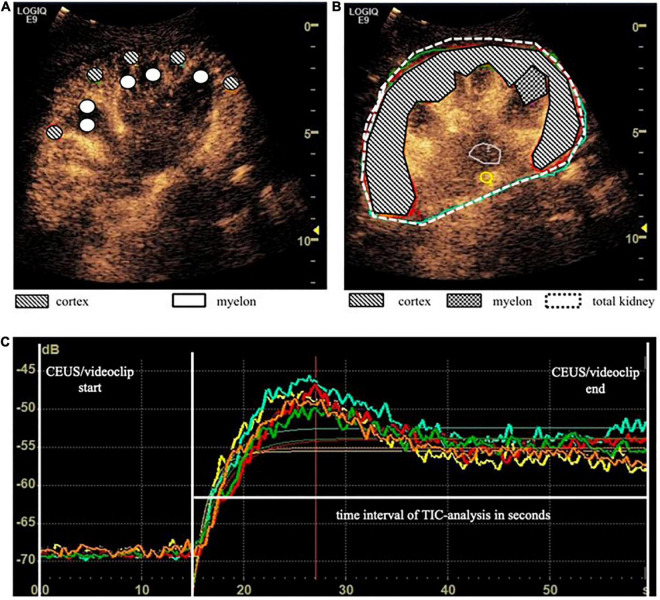
Time-intensity curve analysis (TIC analysis) was calculated based on different region of interest (ROI) methods. **(A)** ROI_5_ and ROI_10_ consisted of 3 to 5 × 5 mm^2^ and 10 mm^2^ placed in the cortex and myelon. TIC parameters of ROI_5/10_ were calculated as averages of the multiple ROIs. **(B)** ROI_*Anat*_ was an anatomical outline of the total kidney, the whole cortex, the upper and the lower cortex, and one representative myelon. **(C)** TIC curves based on ROI_5_ in the cortex.

ROI_5_ and ROI_10_ 3–5 regions were placed in the renal cortex and the myelon, respectively. The shape is circular and has a fixed size of 5 mm^2^ in ROI_5_ and 10 mm^2^ in ROI_10_. TIC parameters of ROI_5_ and ROI_10_ were calculated as averages of the multiple ROIs (Figure 1A).

ROI_*Anat*_ describes the anatomical region (i.e., the total kidney, the whole cortex, the upper and the lower cortex, and one representative myelon). The regions were identified in the B-Mode scan, and the anatomical outline was circumscribed. Therefore, the size of the regions varies from patient to patient but may reflect the size and quality of the transplanted organ (Figure 1B).

The internal device software calculated the intensity-related TIC parameters including A, AUC, Grad [in arbitrary units (a.u.)], and the time-related TIC parameter (TTP) [in seconds (s)] (Figure 1C). A second operator, an experienced CEUS examiner, repeated the 33 TIC analysis with ROI_5_ and ROI_*Anat*_ methods in the cortex and myelon. We investigated the differences and correlations between TIC parameters derived from different ROI methods. Furthermore, we analyzed the impact of ROI depth and the time interval of TIC analysis on TIC parameters and compared the interoperator variance of ROI_5_ and ROI_*Anat*_ between the two operators.

### Statistics

Results were expressed as mean ± *SD* if not indicated otherwise. The differences between groups were compared using the Wilcoxon rank test and the Friedman test (paired samples). The intraclass correlation coefficient was calculated using a two-way mixed model and absolute agreement, and then classification by Koo and Li was applied (19). Pearson correlation analysis determined the relation between TIC parameters and the time interval of TIC analysis. A *p*-value of < 0.05 was considered significant. All data were analyzed using IBM SPSS Statistics version 25.0 (IBM, Armonk, NY, United States).

## Results

### Baseline characteristics

**FIGURE 2:**
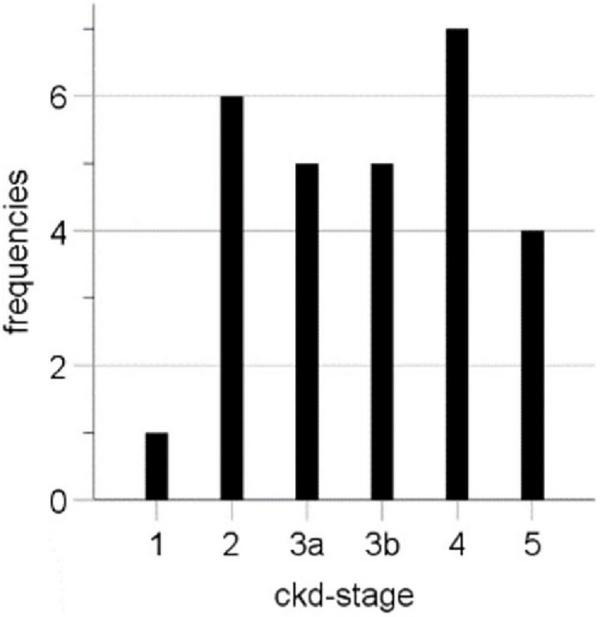
CKD stages at CEUS of examined kidney transplants.

TIC analysis was performed based on 33 CEUS examinations of 25 renal transplants of different patients in the Department of Nephrology at the University Hospital Regensburg. Since the examination of the transplanted kidney was often carried out as part of ultrasound follow-up examinations (e.g., when checking for complicated kidney cysts), it occurred that seven patients received a second CEUS, and one patient received a third CEUS. The average patient age was 54.73 ± 13.66 years (22–79 years), and the majority were men (64%, 16 cases), and the average age of kidney transplant at CEUS was 5.18 ± 4.86 years (0.0–249 months). In 28 cases, laboratory data were available at the time point of the CEUS with a mean creatinine level of 2.53 ± 1.59 mg/dl and a mean eGFR (CKD-EPI) of 40.04 ± 25.28 ml/min/1.73 m^2^. For the CEUS examination, we included patients of all CKD stages (Table 1 and Figure 2).

**TABLE 1:** Patient baseline characteristics.

CEUS—*n*	33
Male—n (%)	16 (64%)
Patient age—years	54.73 ± 13.66
Kidney transplant age—years	5.18 ± 4.86
eGFR at CEUS—ml/min/1,73 m^2^	37,0 ± 23,0
Serum creatinine level—mg/dl	2.53 ± 1.59

### Influence of size and form of region of interest

First, we compared the TIC parameters of all methods, and most frequently, differences showed up between ROI_Anat_ and ROI_10_. In the myelon the differences between ROI_5_ and ROI_10_ were significant but in view of the measured values, the difference was rather low with a deviation of the mean < 10% (ΔA = −1.24 ± 0.55 a.u.; ΔAUC = 29.98 ± 15.44 a.u.; *p* < 0.05), and the ICC remained high in cortex and myelon. Then, we compared the ROIs with fixed surface area (ROI_5_ and ROI_10_) to the ROI_Anat_ method and found variations for TIC parameter Grad and AUC in the cortex and myelon. The ICC between ROI_5_ and ROI_Anat_ decreased in the myelon for parameters A, TTP, and Grad, and if one considered not solely the IC coefficient but also the 95% confidence interval of ICC, the agreement between the two methods must be interpreted as bad (*p* < 0.05). Notably, ROI_5_ was the only method that measured differences between the cortex and myelon for all TIC parameters (Tables 2–4).

**TABLE 2:** Differences in time-intensity curve (TIC) parameter between ROI_5_, ROI_10_, and ROI_Anat_.

	ROI_Anat_	ROI_5_	ROI_10_	*P*-value		
				1	2	3
**Cortex**						
A	20.94 ± 6.11	20.35 ± 5.87	20.99 ± 6.86	0.396	0.432	0.574
TTP	15.12 ± 6.11	14.55 ± 5.19	15.45 ± 7.11	0.177	0.550	0.526
AUC	620.60 ± 294.99	589.95 ± 278.88	564.19 ± 312.39	0.189	0.098	0.026*
Grad	1.44 ± 0.66	1.61 ± 0.66	1.54 ± 0.7	0.001*	0.191	0.145
**Myelon**						
A	18.90 ± 7.63	19.07 ± 6.04	20.31 ± 6.59	0.755	0.025*	0.145
TTP	20.55 ± 7.67	20.55 ± 7.67	19.56 ± 7.78	0.728	0.280	0.782
AUC	502.37 ± 284.65	532.27 ± 292.62	562.18 ± 308.06	0.339	0.014*	0.008*
Grad	0.97 ± 0.44	1.09 ± 0.50	1.20 ± 0.65	0.118	0.095	0.019*

p-value group: 1 = ROI_5_ vs. ROI_Anat_, 2 = ROI_5_ vs. ROI_10_, and 3 = ROI_10_ vs. ROI_Anat_ (n = 33). A, AUC, Grad in a.u.; TTP in seconds.

* *p* < 0.05.

**TABLE 3:** Intraclass correlation of ROI_5_ and ROI_Anat_ and ROI_5_ and ROI_10_.

	ROI_5_ vs. ROI_Anat_		ROI_5_ vs. ROI_10_	
	ICC (95%–CI)	*P*-value	ICC (95%–CI)	*P*-value
**Cortex**				
A	0.873 (0.745–0.937)	0.000	0.887 (0.772–0.944)	0.000
TTP	0.939 (0.876–0.970)	0.000	0.878 (0.754–0.939)	0.000
AUC	0.958 (0.916–0.979)	0.000	0.972 (0.943–0.986)	0.000
Grad	0.951 (0.831–0.981)	0.000	0.931 (0.862–0.966)	0.000
**Myelon**				
A	0.679 (0.344–0.842)	0.001	0.928 (0.844–0.965)	0.000
TTP	0.578 (0.139–0.793)	0.009	0.859 (0.716–0.930)	0.000
AUC	0.941 (0.882–0.971)	0.000	0.983 (0.962–0.992)	0.000
Grad	0.757 (0.513–0.879)	0.000	0.881 (0.758–0.941)	0.000

Intraclass correlation coefficient (ICC) classification: bad < 0.5, moderate 0.5–0.75, good 0.75–0.9, and excellent correlation > 0.9. TIC analysis was performed by operator 1 (n = 33). A, AUC, Grad in a.u.; TTP in seconds.

**TABLE 4:** Differences of TIC parameters between cortex and myelon.

	Cortex	Myelon	*P*-value
**ROI_5_**			
A	20.35	19.07	0.007
TTP	14.55	20.55	0.000
AUC	589.95	532.27	0.001
Grad	1.61	1.09	0.000
**ROI_10_**			
A	20.99	20.31	0.480^#^
TTP	15.45	19.57	0.000
AUC	564.89	562.18	0.600^#^
Grad	1.54	1.20	0.000
**ROI_Anat_**			
A	20.94	18.90	0.098^#^
TTP	15.21	19.89	0.000
AUC	610.61	502.37	0.000
Grad	1.44	0.97	0.000

^#^p > 0.05 (n = 33). A, AUC, Grad in a.u.; TTP in seconds.

### Influence of the localization and depth of the regions of interest

**FIGURE 3:**
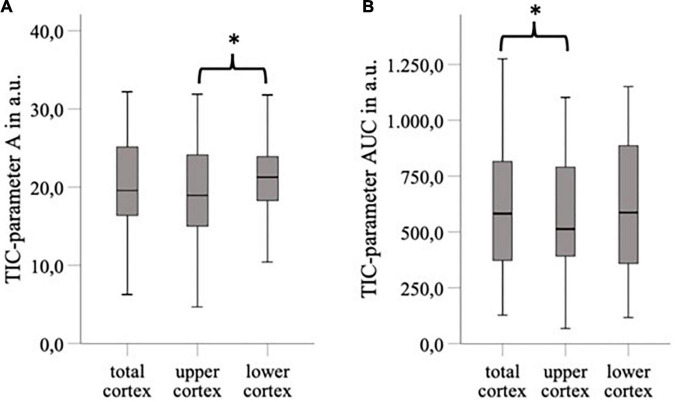
The area of ROI “total cortex” was approximately twice as big as the area of ROI “upper/lower cortex,” and the ROI “lower cortex” and “total cortex” were placed on average 4.44 cm deeper than the “upper cortex” (*p* < 0.05). For TIC parameter, A was a significant difference between “upper cortex” vs. “lower cortex” (ΔA = 2,15 a.u., *p* < 0.05) **(A)** and for TIC parameter AUC between “upper cortex” and “total cortex” (ΔAUC = 39,21 a.u., *p* < 0.05) **(B)**. *p < 0.05.

We investigated the influence of depth of ROI on the TIC parameters. The standardized 5 mm^2^ ROIs no. 1–5 were placed in different regions of the cortex and ROI no. 5 was on average 1 cm deeper than ROI no. 1 (3.5 ± 1.3 cm vs. 4.5 ± 1.7 cm). Nevertheless, the TIC parameters derived by ROI no. 1–5 did not show significant differences (Table 5). Using the ROI_Anat_ method, we investigated differences between ROI “upper/lower/total cortex.” The intensity parameters A and AUC were higher, and the TTP was prolonged in “lower” and “total cortex” vs. “upper cortex.” There were no differences for TIC parameter Grad (Figure 3 and Table 6).

**TABLE 5:** In ROI_5_, variation of ROIs in depth does not affect TIC parameter values.

	nr. 1	nr. 5	*P*-value
Depth in cm	**3.5 ± 1.3**	**4.5 ± 1.7**	0.014*
A	20.86 ± 6.54	20.28 ± 6.31	0.875
TTP	14.08 ± 5.94	14.49 ± 4.82	0.652
AUC	598.95 ± 291.21	611.00 ± 273.59	0.597
Grad	1.65 ± 0.67	1.6 ± 0.70	0.984

Five regions of interest (ROIs) sized 5 mm^2^ were placed in the cortex at different distances from the ultrasound probe. The value “depths in cm” describes the distance between ROI in the parenchyma and the ultrasound probe measured in cm. The TIC parameters derived by ROI no. 1 did not differ significantly from TIC parameters derived by ROI no. 5 (Friedman test, p > 0.05), though ROI no. 5 was localized on average 1.05 cm deeper in the cortex than ROI no. 1 (p < 0.05). N = 31 (in two TIC analyses, just four ROIs could be placed sufficiently). A, AUC, Grad in a.u.; TTP in seconds.

*p < 0.05.

**TABLE 6:** Impact of depths in ROI_Anat_ on TIC parameter values in the cortex.

	Cortex			*P*-value		
	Total	Upper	Lower	1	2	3
Depth in cm	8.33 + 1.79	3.48 + 0.96	7.50 + 1.30	0.000*	0.000*	0.003*
A	20.94 + 6.11	19.54 + 5.99	21.69 + 5.33	0.055	0.014*	0.313
TTP	15.21 + 6.11	14.47 + 4.44	17.57 + 10.09	0.147	0.140	0.161
AUC	610.61 + 294.99	571.40 + 272.72	613.82 + 302.25	0.024*	0.091	0.574
Grad	1.44 + 0.66	1.48 + 0.70	1.36 + 0.69	0.755	0.304	0.416

p-value group: 1 = upper vs. total. 2 = upper vs. lower 3 = total vs. lower; A, AUC, Grad in a.u.; TTP in seconds.

*p < 0.05.

### Interoperator variance

**FIGURE 4:**
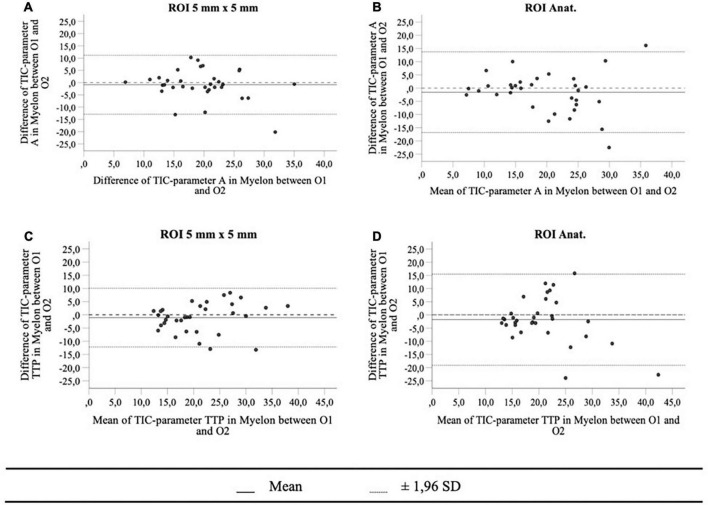
The Bland-Altman plots for TIC parameters A **(A,B)** and TTP in myelon **(C,D)** show a greater level of agreement (mean ± 1,96 *SD*) for method ROI_Anat_ than for ROI_5_.

We investigated the interoperator variance of TIC analysis between two operators using ROI_5_ and ROI_Anat_ methods. Apart from the TIC parameter Grad, which showed a slight bias of 0.14 between operators 1 and 2 in the myelon, there were no significant differences between the two operators (Table 7). Yet, in the myelon, the deviation between the two operators increased compared with the cortex and was generally higher with ROI_Anat_ than with ROI_5_ (Table 8). The higher interoperator variance for method ROI_Anat_ is especially reflected in a greater level of agreement (LoA) in the myelon for parameters A and TTP (Table 9 and Figure 4).

**TABLE 7:** Differences of TIC parameters between operators 1 and 2.

	ROI_5_			ROI_Anat_		
	O1	O2	*P*-value	O1	O2	*P*-value
**Cortex**						
A	20.53 ± 5.8	20.46 ± 6.72	0.492	20.94 ± 6.11	23.09 ± 8.21	0.067
TTP	14.55 ± 5.19	15.83 ± 6.55	0.088	15.12 ± 6.11	25.52 ± 6.43	0.911
AUC	589.95 ±	573.54 ±	0.067	610.62 ±	655.14 ±	0.210
	277.87	300.44		294.99	295.38	
Grad	1.61 ± 0.66	1.50 ± 0.71	0.085	1.44 ± 0.66	1.45 ± 0.68	0.501
**Myelon**						
A	19.07 ± 6.04	19.92 ± 7.35	0.427	18.90 ± 7.63	20.50 ± 8.68	0.313
TTP	20.55 ± 7.67	21.63 ± 6.74	0.480	19.89 ± 6.50	21.66 ± 8.74	0.166
AUC	532.27 ±	535.76 ±	0.102	502.37 ±	538.71 ±	0.837
	292.62	312.42		284.65	326.02	
Grad	1.09 ± 0.50	0.95 ± 0.36	0.013*	0.97 ± 0.44	0.91 ± 0.36	0.503

Wilcoxon rank test, *p < 0.05, n = 33. O1 = operator 1, O2 = operator 2; A, AUC, Grad in a.u.; TTP in seconds.

**TABLE 8:** Intraclass correlation between TIC parameters of operators 1 and 2.

	ROI_5_		ROI_Anat_	
	ICC (95%–CI)	*P*-value	ICC (95%–CI)	*P*-value
**Cortex**				
A	0.915 (0.828–0.958)	0.000	0.579 (0.162–0.791)	0.007
TTP	0.834 (0.665–0.918)	0.000	0.903 (0.802–0.953)	0.000
AUC	0.922 (0.843–0.961)	0.000	0.929 (0.855–0.965)	0.000
Grad	0.917 (0.829–0.959)	0.000	0.952 (0.902–0.977)	0.000
**Myleon**				
A	0.738 (0.471–0.871)	0.000	0.717 (0.433–0.859)	0.000
TTP	0.824 (0.648–0.913)	0.000	0.543 (0.087–0.773)	0.014
AUC	0.879 (0.754–0.940)	0.000	0.880 (0.758–0.940)	0.000
Grad	0.752 (0.498–0.877)	0.000	0.701 (0.397–0.852)	0.001

Intraclass correlation coefficient classification: bad < 0.5, moderate 0.5–0.75, good 0.75–0.9, excellent correlation > 0.9; A, AUC, Grad in a.u.; TTP in second.

**TABLE 9:** Bland Altman statistics for ROI_5_ and ROI_Anat_.

	ROI_5_		ROI_Anat_	
	LoA (bias ± 1.96*SD)	*P*-value	LoA (bias ± 1.96*SD)	*P*-value
**Cortex**				
A	−0.11 ± 7.00	0,863	−2.38 ± 15.19	0.091
TTP	−1.28 ± 8.57	0.103	−0.21 ± 7.41	0.761
AUC	16.40 ± 307.92	0.552	−30.98 ± 294.05	0.253
Grad	0.12 ± 0.72	0.077	−0.02 ± 0.57	0.710
**Myelon**				
A	−0.85 ± 12.03	0.432	−1.60 ± 15.00	0.238
TTP	−1.08 ± 10.92	0.273	−1.76 ± 16.87	0.248
AUC	−3.49 ± 394.29	0.921	−36.35 ± 392.80	0.305
Grad	0.14 ± 0.70	0.044*	0.06 ± 0.75	0.378

p-value refers to bias (*p < 0.05); A, AUC, Grad in a.u.; TTP in seconds.

### Influence of the time interval of the cine-loop

As TIC analysis was carried out retrospectively, the duration of CEUS video clips available for TIC analysis differed in some cases and resulted in a variation in time. This is due to the fact of slightly different circulation times between the patients. The mean time interval used for TIC analysis was 47.31 ± 15.18 s, and Table 10 shows a strong correlation between the time interval of TIC analysis and the TIC parameter AUC in the cortex and myelon (*r* = 0.710 and 0.674, *p* < 0.000). Compared with the correlation between AUC and the time interval, the correlation between TTP and time interval was not significant (*r* = 0.389, *p* > 0.05), and TIC parameters A and Grad did not correlate with the time interval at all.

**TABLE 10:** Pearson correlation coefficient between the time interval of TIC analysis and TIC parameters.

	Correlation coefficient, *r* (*P*-value)			
	A	TTP	AUC	Grad
Cortex	−0.257 (0.149)	−0.118 (0.513)	0.710 (0.000*)	0.287 (0.105)
Myelon	−0.225 (0.208)	0.389 (0.025*)	0.674 (0.000*)	−0.038 (0.833)

r > 0.5 is considered a strong correlation, *p < 0.05; A, AUC, Grad in a.u.; TTP in second.

## Discussion

TIC analysis of kidney transplants is a promising field of research to detect early signs of organ dysfunction through reduced microperfusion, especially in the cortical region of the kidney transplant. Unfortunately, to date, there is no standardized protocol to measure the different TIC parameters in organs with different compartments, e.g., transplanted kidneys. In this study, we tried to determine the factors, which influence the value of TIC parameter analysis in kidney transplants.

In general, the ROI should be large enough to also allow the detection of heterogeneous perfusion signs (20, 21). To date, there is no standardized protocol for the size or form of ROI for TIC analysis in kidney transplants resulting in an inhomogeneous use of ROI mainly sized 5 or 10 mm^2^ (22–26) of an anatomical outline (27–32) or was clearly not indicated (33–36). Table 11 gives an overview of the localization, size, and form of ROIs of various studies with CEUS in kidneys. Leinonen et al. reported an inverse correlation between the size of the ROI and the intensity parameters (37). This goes along with our results, suggesting size impacts, especially TIC parameters representing the signal intensity like A and AUC. We recommend using a size of 5 mm^2^ for various reasons. First, placement of up to five ROIs in cortex and myelon was in most cases possible with an ROI of 5 mm^2^. In comparison, with 10 mm^2^ in some cases, only three ROIs could be positioned, as the thin cortex did not allow the exact placement without including other structures, e.g., the medulla or vascular structures, and correct placement of ROI with 10 mm^2^ size was more time-consuming than the positioning of 5 mm^2^. Second, a greater surface of ROI makes it more likely to include vascular structures, e.g., AA. interlobares, AA. arcuatae, and AA. interlobularis in unnoticed manner, which should be avoided in the analysis, as this distorts the perfusion analysis of microcirculation (18, 37). The arteries show a faster and increased contrast enhancement, which then leads to significant changes in the TIC parameters in the ROIs. This could also be seen in our analysis. TIC parameters representing the signal intensity did not differ significantly in the cortex or the myelon by using ROI_10_ (area 10 mm^2^) probably because other anatomical structures were included in the area of 10 mm^2^.

**TABLE 11:** Comparison of different ROI-sizes and -forms used for the TIC-analysis in kidney transplants in different studies.

References	ROI form	ROI location	Number of ROIs per region	US-device	Software	Kinetics of CEUS	Aim of study	Study size
Wang et al. (22)	Square	Cortex; myelon;	1;1;	IU 22 (Philips)	QLAB (Philips)	Bolus	Evaluate perfusion parameters 1–6 months after transplantation	35
Yoon et al. (23)	Square	Cortex; myelon;	3;3;	IU 22 (Philips)	QLAB (Philips)	Bolus	Evaluate CEUS-parameters as predictors of outcome in acute kidney injury	48
Liang et al. (24)	Circular	Cortex; myelon; interlobar artery; segmental artery	1;1;1;1;	IU 22 (Philips)	Sonoliver (TomTec Imaging Systems)	Bolus	Evaluate CEUS in the assessment of renal allograft dysfunction	57
Cai et al. (25)	Circular	Cortex	2;	GE LOGIQ 9 (GE Healthcare)	Device internal software	Bolus	Compare TIC-parameters between normal graft and delayed graft function	44
Jin et al. (26)	Circular	Cortex	2;	GE LOGIQ 9 (GE Healthcare)	Device internal software	Bolus	Reliability of CEUS on the diagnosis of acute (AR) or chronic rejection (CR) after renal transplantation	79
Álvarez Rodríguez et al. (33)	Circular (no size)	Cortex; myelon, interlobar artery	1;	–	–	Bolus	Assess the effectiveness of CEUS in the early post-transplant period of kidneys	15
Benozzi et al. (34)	Circular (no size)	Cortex; corticomedullary axis;	2; 2;	–	–	Bolus	Compare CEUS to doppler-US in detection of early graft dysfunction	39
Fischer et al. (35)	Circular (no size)	Main artery; cortex; renal vein;	1;1;1;	Aplio (Toshiba)	Device internal software	Bolus	Evaluate kidney recipients in the early posttransplant phase by TIC-analysis	22
Fischer et al. (36)	Circular (no size)	Main artery; interlobar artery; cortex; renal vein;	1;1;1;1;	Aplio (Toshiba)	Device internal software	Bolus	Determine the value of CEUS in the assessment of early allograft dysfunction	45
Schwenger et al. (27)	Outline of the region	Cortex	1;	ATL HDI 5000 (Philips)	QLAB (Philips)	Flash replenishment	Feasibility of CEUS detecting CAN in comparison to color doppler US	26
Araújo and Suassuna (28)	Outline of the region	Cortex; myelon; segmental artery;	1;1;1;	Aplio 400 (Toshiba)	Device internal software	Bolus	Differences of TIC-analysis between early and late graft dysfunction	67
Brabrand et al. (29)	Outline of the region	Cortex; myelon;	1; 1;	Acuson Sequoia 512 (Siemens)	nordicICE; nordic imaging lab	Bolus	Evaluate changes in perfusion with CEUS due to global hypoxia in piglets	12
Jeong et al. (30)	Outline of the region	Cortex	1;	RS80A (Samsung Medison)	VueBox^§^; Bracco	Bolus	Evaluate clinical significance of CEUS in CKD	24
Stock et al. (31)	Outline of the region	Cortex; myelon; interlobar artery	3;2;1;	IU 22 (Philips)	VueBox^§^, Bracco	Bolus	Evaluate renal perfusion with CEUS in cats with CKD	57
Kihm et al. (32)	Outline of the region	Cortex	1;	ATL TDI 5000 (Philips)	QLAB	Flash replenishment	Evaluate change in microperfusion due to ciclosporine A and tacrolimus by CEUS	32

The next question was whether it is necessary to include an entire anatomical region within the TIC analysis or only a representative, preformed area within this region. The rationale for using anatomic ROIs was that in standardized sections of the transplanted kidney, the size and configuration of the anatomic region could also provide an additional indication of the future renal function, which cannot be provided by single standardized sections of the anatomic region. When it comes to the form of ROI, a preformed size of 5 mm^2^ offers more standardization than a freehand drawn outline of the anatomic region (ROI_Anat_). The high variation of TIC parameters between ROI_5_ and ROI_Anat_ shows that both methods could not be used as equivalent. To decide on one of the two methods, we included the following aspects into consideration: first, the variation of TIC parameters especially within the myeloid structures between the ROI_5_ and ROI_Anat_ could be explained by the non-myeloid structure being unintentionally included in the freehand drawn ROI. This is supported by the higher interoperator variance between operators 1 and 2 in the myelon for ROI_Anat_. Second, the area of ROI_Anat_ varied, whereas the area of ROI_5_ was constant. As Leinonen et al. reported, an equal area of ROI is a necessary criterion for constant TIC analysis (37). So far, there is no literature that distinguished these two methods before, but our results recommend an application of multiple ROIs sized 5 mm^2^ for further TIC analysis.

Using ROI_5_, the average difference in depth between the single ROI with 5 mm^2^ was solely 1.0 cm and did not result in different TIC parameters. In contrast to that, with ROI_Anat_, there was an average difference of 4.5 cm that led to differences in TIC parameters. The method ROI_Anat_ showed that not the size of ROI, but predominantly the depth of ROI influenced the values of intensity-related (A, AUC) and time-related TTP. It is up to the technique of ultrasound itself that signal attenuation correlates with distance to the ultrasound probe and may reflect in different values of TIC parameters (37). Nevertheless, with ROI_5_, we recommended placing the ROIs in well-perfused and distinct regions that are representative of the anatomic region and handle depth as a secondary criterion for the location of ROIs.

The CEUS examination should be performed only by experienced investigators (12, 38) and yet the performance and subsequent assessment of the CEUS examination are highly examiner-dependent. The most important thing to mention in this study is that the examination is carried out without movement and without pressure on the graft. Regardless of this, the TIC analysis allows objective quantification of perfusion separately from the CEUS examination. In this study, we analyzed the interoperator variance of TIC analysis between two investigators for ROI_5_ and ROI_Anat_. Overall, the agreement of TIC analysis between investigators 1 and 2 was high but in comparison to the cortex, the agreement decreased in the myelon. This is remarkable because although both investigators had different levels of experience, the results were consistent, despite the fact that renal tissue is very inhomogeneous, and different compartments were measured separately. Our results are supported by Nylund et al. who also found a low interoperator variance of TIC analysis with inflammatory bowel disease (39). We preferred the standardized 5 mm^2^ form ROI_5_ instead of the anatomic form ROI_Anat_. For ROI_Anat_, the interoperator variance for the parameters A and TTP was so high that the clinical application is not reasonable and the method ROI_5_ should be preferred.

TIC analyses were performed retrospectively after CEUS examination and consequently, the cine-loops lasted in some cases less than 60 s and led to a variation of the time interval for TIC analysis with a mean of 47.31 ± 15.18 s. This is due to the fact that in some patients, the arrival time in the kidney transplant was longer than in others, and the cine-loops were standardized to a length of 60 s after contrast-agent application. But this allowed us to determine the influence of the time interval of the cine-loop on the different TIC parameters. Our results showed a strong correlation between the time interval and the TIC parameter AUC. Many authors emphasize the use of AUC in the clinical context (40, 41), but if the TIC parameter is dependent on the time interval, its informative value is limited. Therefore, our results emphasize the need for a standardized start and endpoint of TIC analysis to generate a consistent time interval for TIC analysis. To date in many studies, there is no standardized length of the video clip, but this is crucial to define clear results and cutoff values of AUC and TTP in future studies.

In general, the time interval of the TIC analysis should include the contrast agent wash-in phase and representative parts of the wash-out phase. The entire wash-out phase of the contrast agent may take up to 10 min in the bolus model (18, 42), and integration of the entire wash-out phase into the TIC analysis would be too time-consuming, not practical, and inappropriate for the patient examination. With a view to a uniform time interval, the stop setting needs to be further evaluated in follow-up studies. An approach following Kay et al. would be conceivable. The authors normalized the time interval to 5 s after initiation of the contrast agent and described a correlation of AUC with eGFR 3 months after renal transplantation (43). Other experimental approaches would be a stop point 30 s after the arrival of the contrast agent to capture the cortical phase or after 60 s to capture portions of the medullary phase (12, 44).

The main limitation of this study is the limited number of subjects, and the results should be confirmed in a larger population. However, this study should generate hypotheses that should be tested in a larger cohort in a clinical context. In our study, we applied “Wash-in” kinetics as it best represents the perfusion. Eventually, patients with hyperdynamic circulation who show an early wash-out might lead to a bias in the TIC parameters. If extreme abnormalities in the visual evaluation of the perfusion kinetics were referred to as measuring errors, these CEUS examinations were excluded from the study. For the assessment of interoperator variance, the LoA has to be discussed in a clinical context (45). Consequently, till present, the assessment of interoperator variance is limited due to the lack of a generally applicable value range for TIC analysis with kidney transplants. Furthermore, no clinical parameters were included in this study, but this has to be the subject of further studies after the examination has been standardized.

## Conclusion

Identifying kidney transplant recipients at increased risk for graft failure is one of the most important tasks in transplant medicine. TIC analysis could make a key contribution to improving long-term graft survival. But before TIC parameters can be used to define threshold values for good or limited future graft function, the procedure of TIC analysis should be standardized because TIC parameters are influenced by various factors. We recommended the use of an average of multiple ROIs of 5 mm^2^ in the cortex and myelon. The method of ROI 5 mm^2^ offers a standardized form and a sufficient, feasible size, which enables TIC analysis with low intraoperator and interoperator variance. The duration of the video clip should be set at 60 s after the contrast agent has reached the kidney transplant. With regard to further improvement of TIC analysis in kidney transplants, we emphasized concluding with one standardized method of ROI.

## Data availability statement

The raw data supporting the conclusions of this article will be made available by the authors, without undue reservation.

## Ethics statement

The studies involving human participants were reviewed and approved by the Ethikkomitee Universitätsklinik Regensburg. Written informed consent for participation was not required for this study in accordance with the national legislation and the institutional requirements.

## Author contributions

SF collected, analyzed the data, wrote, reviewed, and edited the manuscript. FP conceptualized the study design, performed the CEUS examination, and edited and reviewed the manuscript. SF, EJ, TB, HT, MB, BB, and FP contributed to manuscript revision and read and approved the final version. All authors contributed to the article and approved the submitted version.
